# Heat shock factor 2-binding protein promotes tumor progression via activation of MAPK signaling pathway in lung adenocarcinoma

**DOI:** 10.1080/21655979.2022.2063561

**Published:** 2022-04-17

**Authors:** Junyuan Liu, Yuting Zhang, Jie Tao, Tingting Yu, Tao Zhang

**Affiliations:** a Department of Thoracic Oncology, Xinjiang Medical University Affiliated Tumor Hospital, Urumqi, Xinjiang, China; b Department of Geriatrics, The Fifth Clinical Medical College of Xinjiang Medical University, Urumqi, Xinjiang China; c Department of Oncology, The First Hospital of Lanzhou University, Lanzhou, China

**Keywords:** HSF2BP, lung adenocarcinoma, prognosis, MAPK signaling pathway

## Abstract

Lung adenocarcinoma (LUAD) is a malignant tumor that causes a serious public health burden. The biological functions and potential mechanism of heat shock factor 2-binding protein (HSF2BP) in LUAD have not been studied. This study aimed to explore the HSF2BP expression pattern and its potential biological function in LUAD. The transcriptome data and relevant clinical data of LUAD were downloaded from The Cancer Genome Atlas (TCGA) database. The mRNA levels and prognosis of HSF2BP were determined using TCGA datasets. The protein and mRNA expression levels of HSF2BP were identified by conducting western blot analysis and quantitative real-time polymerase chain reaction in tissues and cells, respectively. To determine whether HSF2BP affected the biological function of LUAD cell lines, a series of functional experiments were performed in vitro and in vivo. In addition, gene set enrichment analysis was applied to determine the pathways that HSF2BP regulated, which was further confirmed by western blotting, and the high expression of HSF2BP was observed in LUAD, which was correlated with the unfavorable prognosis in LUAD patients. Clinical correlation analysis revealed that tumor stage was positively correlated with high HSF2BP expression. Furthermore, HSF2BP could serve as an independent risk factor for overall survival. In vitro, HSF2BP knockdown suppressed the proliferation and migration of A549 and H1299 cells. We observed the same results in vivo experiments. Mechanistically, the HSF2BP regulates the mitogen-activated protein kinase signaling pathway to perform its biological function. The HSF2BP plays a role in the development of LUAD and could be a useful anticancer target for the treatment of LUAD.

## 1. Introduction

According to the GLOBOCAN 2020 Cancer Incidence Statistics, lung cancer remains the most common cause of cancer-related deaths [1]. Non-small cell lung cancer is the primary component of lung cancer, of which lung adenocarcinoma (LUAD) is the most frequently diagnosed histological subtype [2–4]. Despite the significant advances in the molecular targeted therapy of LUAD, the overall survival of patients with LUAD remains grim [5]. With the development of bioinformatics, more targets are being identified [6,7]. Therefore, exploring the effective targeted treatment regimens and identifying the potential biomarkers are essential to improve the prognosis of patients with LUAD.

The heterotopic expression and activation of germline genes have a fundamental effect on the cancer onset, which results in genomic instability [8,9]. The improper repair of DNA damage, which leads to genomic instability, and homologous recombination (HR) pathway repair are crucial for maintaining the genomic integrity and preventing tumor development [10,11]. Heat shock factor 2-binding protein (HSF2BP) was initially reported to encode the interaction factor of heat shock response transcription factor 2 [12]. It is a germ-line-specific protein that affects the spermatogenesis by regulating the BNC1 transcriptional activity and subcellular localization [13]. HSF2BP, a major regulator of meiotic recombinase, affects the recombinase location and interferes with DNA double-strand break repair defects and subsequent dysplasia during HR repair [14]. In addition, BRCA2 acts as a tumor suppressor in mitotic HR, and HSF2BP can bind to BRCA2 and mediate its recombination localization, thereby promoting cancer development by interfering with the mitotic HR pathway [15]. However, the expression and role of HSF2BP in LUAD need to be investigated further.

Given that HSF2BP plays a critical role in genomic instability, we infer that it may contribute to the progression of malignancies. In this study, we aimed to investigate the HSF2BP expression and function via a series of functional assays in LUAD and explore its underlying downstream mechanism. To the best of our knowledge, we reported for the first time that HSF2BP was elevated in the LUAD and contributed to the progression of LUAD. Furthermore, it might be involved in the regulation of mitogen-activated protein kinase (MAPK) signaling pathway.

## 2. Materials and methods

### 2.1. Bioinformatics

Based on the TCGA database, the expression data of 59 normal samples and 535 LUAD samples were obtained to further analyze the HSF2BP mRNA expression levels. Then, the ‘survival’ package was used for survival analysis of HSF2BP. The correlation between HSF2BP expression and the clinicopathological factors was evaluated. Cox regression analysis was conducted to determine the independent prognostic value of HSF2BP. GSEA was performed to explore the potential enrichment of the signaling pathways for HSF2BP.

### 2.2. Patients and tissue samples

In total, 20 pairs of fresh LUAD and matching paracancer tissues were collected from LUAD patients who underwent surgery at Xinjiang Medical University Affiliated Tumor Hospital. These patients did not receive chemotherapy or radiation therapy prior to surgery. Before collecting and analyzing the samples, all patients included in the study signed a written informed consent. This study was approved by the ethics committee of Xinjiang Medical University Affiliated Tumor Hospital (No. G-2020003).

### 2.3. Cell lines culture

LUAD cell lines (NCI-H1299, NCI-H209, A549, and NCI-H524), combined with BEAS-2B, which is a human normal lung epithelial cell line, were purchased from the Cell Bank of the Chinese Academy of Sciences (Beijing, China). The Dulbecco’s Modified Eagle Medium containing 10% fetal bovine serum (FBS; Gibco, Grand Island, NY, USA) was used for cell culture at 37°C and 5% CO_2_.

### 2.4. Cell transfection

Lentivirus for short-hairpin RNA downregulating HSF2BP (sh-HSF2BP) was obtained from GenePharma (Shanghai, China). A blank lentivirus vector was used as a negative control (shCtrl). After 72 h of transfection, the stable cell lines were screened with 1 mg/ml of puromycin for 3 days at 37°C.

### 2.5. CCK-8 assay

To assess for cell proliferation, the Cell Counting Kit (CCK)-8 (Bevotime, Shanghai, China) assay was performed in A549 and H1299 cells. After transfection, the cells with a density of 1 × 10^4^ cells/well were seeded following the standard procedure. At 0 h, 24 h, 48 h, and 72 h, the cells were infiltrated with CCK-8 solution and incubated at 37°C in a constant temperature incubator for 4 h. Then, a microplate reader was set to 450 nm to measure the absorbance value for further analysis.

### 2.6. Clonogenic assay

To evaluate the ability of reproduction, approximately 1 × 10^3^ cells were spread separately on each well of 6-well plates under suitable conditions for 10 days. Afterward, the adherent cells were washed with PBS. Then, 4% paraformaldehyde was used to fix the cell colonies. The colonies were counted and photographed to assess for cell proliferation after staining them with 0.1% crystal violet.

### 2.7. Wound healing assay

To assess for wound healing ability, the cells grown to 90% confluence on a 6-well plate were used for the experiment. A sterile 200-μl pipette tip was used to form a scratch wound in the cell monolayer, and the PBS was gently applied to remove the non-adherent cells. The cells were further cultured in serum-free medium to assess for scratches at specific time points.

### 2.8. Transwell assay

Cell migration assays were performed using 24-well plates and transwell chambers. In the upper chamber, 5 × 10^4^ cells resuspended in serum-free medium were added. Meanwhile, a medium containing FBS (10%) was added to the lower chamber. At 24 h, the medium in the upper chamber was removed, and the noninvasive cells were wiped clean using a cotton swab. After fixing with 4% paraformaldehyde, the cells that migrated to the lower chamber were stained with 0.1% crystal violet to determine the number of cells.

### 2.9. Quantitative real-time PCR (qRT-PCR)

Total RNA was extracted from tissues using a TRIzol reagent (Invitrogen, Grand Island, NY, USA), and cDNA synthesis was conducted using the reverse transcription kit (Takara Bio, Shiga, Japan). The relative expression was detected using SYBR Green PCR Master Mix (Takara Bio, Shiga, Japan) on a Bio-Rad CFX96GRT-PCR system. The relative quantification was calculated using the 2^−ΔΔCt^ cycle threshold method. The primer sequences are listed in Table S1.

### 2.10. Western blot

The total proteins of tissues and cell samples were extracted using a radioimmunoprecipitation assay buffer (Solarbio. Equal amounts of protein (20 μg) were separated by sodium dodecyl sulfate–, Beijing, China)polyacrylamide gel electrophoresis and then transferred to polyvinylidene fluoride membranes (Millipore, MA, USA). After blocking with 5% bovine serum in TBST, the membranes and corresponding primary antibodies were incubated overnight in a shaker at 4°C. The primary antibodies used were as follows: anti-GAPDH (1:10,000, ProteinTech, Wuhan, China), anti-HSF2BP (1:1000, Abcam, USA), ERK (1:500, Abcam, USA), phospho-ERK (1:500, Abcam, USA), JNK (1:1,000, Abcam, USA), phospho-JNK (1:1,000, Abcam, USA), p38 (1:1,000, Abcam, USA), and phospho-p38 (1:1,000, Abcam, USA). After incubation with horseradish peroxidase-conjugated secondary antibodies (1:10,000, ProteinTech, Wuhan, China) at room temperature for 1 h, the membranes were visualized using the enhanced chemiluminescence method (Thermo, MA, USA) with a chemiluminescent detection system (Tanon, Shanghai, China).

### 2.11. Immunohistochemistry (IHC)

All the tumor samples were embedded in paraffin and sectioned after fixed with formalin. For IHC, citrate buffer and hydrogen peroxide were used to repair antigen and block endogenous peroxidase respectively. Then sections were blocked with 5% BSA for 20 minutes, which were added with anti-HSF2BP (1: 100, Proteintech, China) at 4°C overnight. Followed by DAB chromogenic solution and hematoxylin, the sections were incubated with the secondary antibody. The percentage of positive cells and intensity were assessed.

### 2.12. Tumor xenograft model

To investigate the role of HSF2BP *in vivo*, we performed xenograft models with the BALB/c nude mice. All mice were raised in a specific pathogen-free environment. A459 cells with sh-HSF2BP and shCtrl were injected subcutaneously into the right side of BALB/c nude mice (six mice per group). The tumor volumes (length×width^2^/2) and weight were measured every 7 days after injection. All experimental procedures were conducted in accordance with the institutional guidelines for the care and use of laboratory animals, and were approved by the Institutional Animal Care and Use Committee of Xinjiang Medical University Affiliated Tumor Hospital (IACUC-20200302-01). One months later, all mice were sacrificed and tumors were removed and collected for hematoxylin and eosin (HE) staining and IHC.

The tumor samples were embedded in paraffin and sectioned after fixed with formalin.For HE staining, the deparaffinized sections were stained with a hematoxylin solution. After dehydration with 70% and 90% alcohol, the sections were stained with eosin. For IHC, citrate buffer and hydrogen peroxide were used to repair antigen and block endogenous peroxidase respectively. Then sections were blocked with 5% BSA for 20 minutes, which were added with anti-Ki67 (1:200, Proteintech, China) at 4°C overnight. Followed by DAB chromogenic solution and hematoxylin, the sections were incubated with the secondary antibody.

### 2.13. Statistical analysis

All data were expressed as average ± standard deviation. SPSS software v17.0 and GraphPad Prism 8.0 were used to perform all statistical analyses. The significant differences between the two groups were assessed using one-way analysis of variance and Student’s *t*-test. A Cox regression analysis was performed to determine the factors affecting the overall survival of LUAD patients, the survival curves were plotted using the Kaplan-Meier method, and the survival differences were evaluated using the log-rank test. A *p* value of <0.05 was considered significant.

## 3. Results

### 3.1. HSF2BP upregulation in human LUAD tissues and its correlation with LUAD stage and unfavorable prognosis

**Figure 1.**
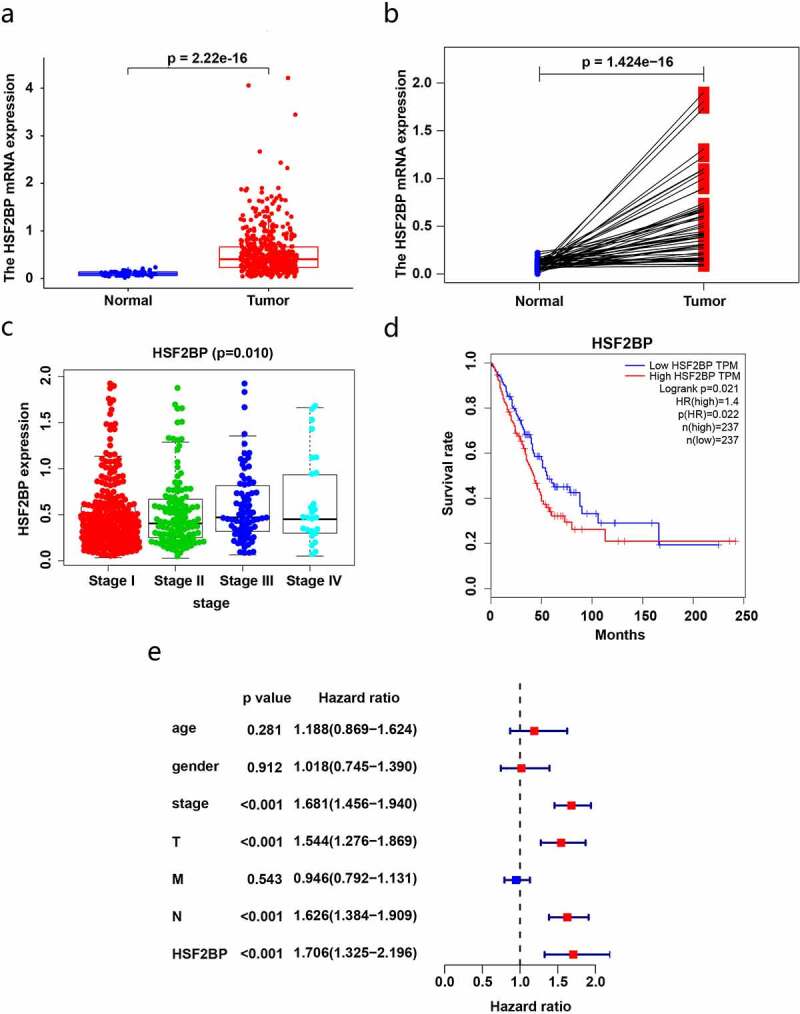
HSF2BP expression and prognosis in LUAD based on TCGA datasets. (a) The mRNA expression in LUAD and normal samples. (b) The expression in LUAD and paired normal samples. (c) Correlation analysis between HSF2BP expression and tumor stage. (d) Survival curves of HSF2BP in LUAD. (e) Cox regression analysis of HSF2BP and clinical parameters.

To investigate the effect of HSF2BP on LUAD, we utilized the TCGA database to assess the HSF2BP mRNA expression and its association with LUAD prognosis. Compared with that in normal tissues, the HSF2BP mRNA levels were markedly higher in the tumor tissues (Figure 1a-b). Combined with a clinical correlation analysis, the HSF2BP expression in LUAD tissues was positively correlated with tumor stage (Figure 1c), but not with other clinicopathological parameters. Based on the TCGA-LUAD dataset, the Kaplan-Meier analysis revealed that a higher HSF2BP expression was correlated with a worse prognosis (Figure 1d). In addition, HSF2BP was an independent prognostic factor according to the results of the Cox regression analysis (Figure 1e).

**Figure 2.**
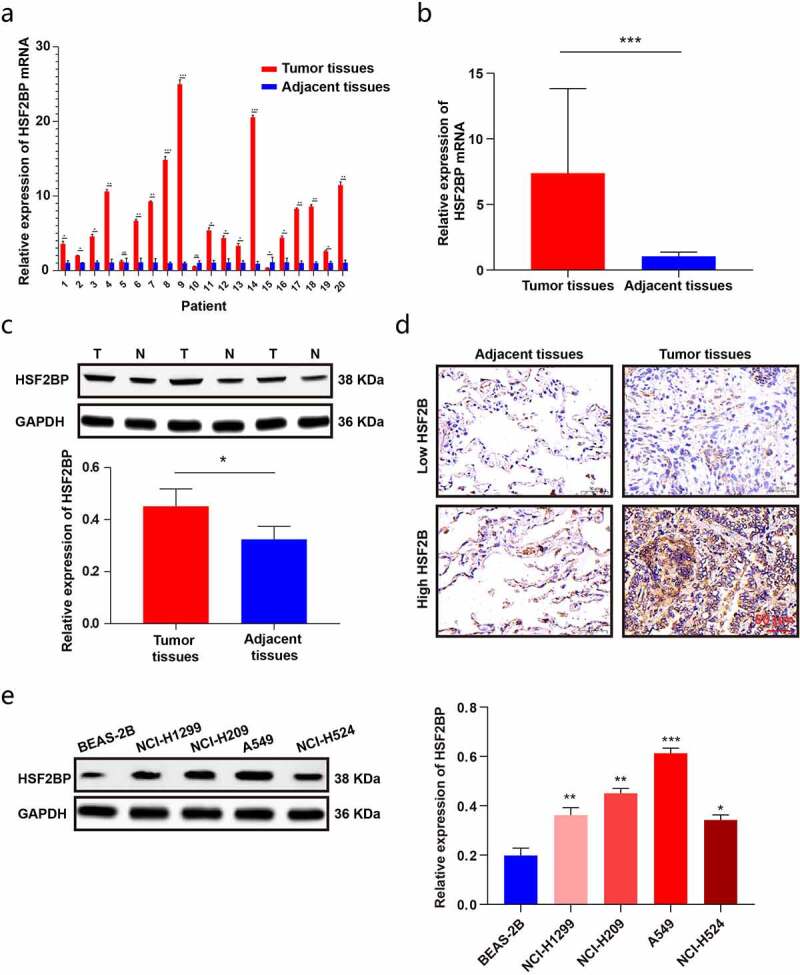
The expression of HSF2BP in LUAD tissues and cells. (a-b) The mRNA expression of HSF2BP in LUAD and normal tissues. (c) The protein expression of HSF2BP in LUAD and adjacent tissues which was detected by western blot. (d) The protein expression of HSF2BP in LUAD and adjacent tissues which was detected by IHC. (e) The protein expression of HSF2BP in LUAD cell lines and BEAS-2B. **Note**: * *p* < 0.05, ** *p* < 0.01, *** *p* < 0.001.

To further validate the expression of HSF2BP in LUAD tissues, the HS2BP expression was evaluated by performing qRT-PCR and a western blot analysis in LAUD tissues. The results were in line with those of TCGA analysis, showing that HSF2BP was overexpressed in LUAD tissues (Figure 2a–c). We further examined the localization of HSF2BP protein by IHC. As shown in Figure 2d, HSF2BP protein was mainly localized in the cytoplasm of cells. In addition, the HSF2BP expression increased in LUAD cell lines compared with that in BEAS-2B cells (Figure 2e). Therefore, the results indicated that HSF2BP might promote the development of LUAD and can be a potential prognostic biomarker.

### 3.2. HSF2BP promoting the proliferation of LUAD cells

**Figure 3.**
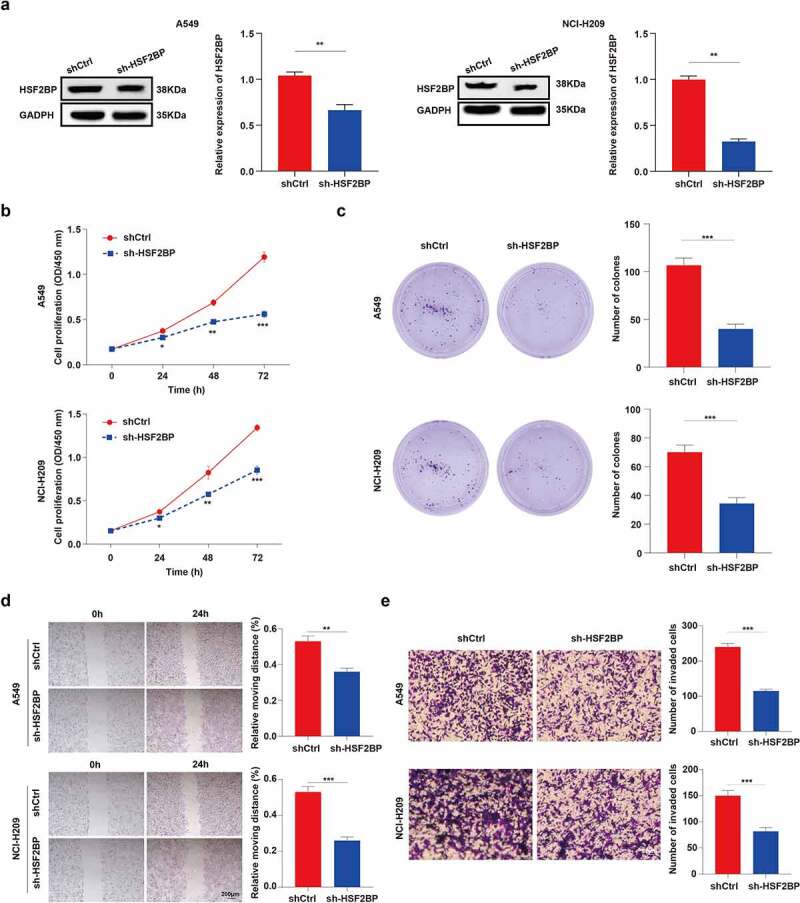
HSF2BP promotes the proliferation and migration of LUAD cells. (a) The transfection efficacy of lentivirus down-regulating HSF2BP in A549 and NCI-H209 cells. (b) The cell viability of A549 and NCI-H209 cells were determined by CCK-8 assay. (c) The colony formation assay was used to measure proliferation in LUAD cells. (d) Wound healing assay in A549 and NCI-H209 cells. (e) Transwell assay was used to determine the migration ability of LUAD cells. **Note**: * *p* > 0.05, ** *p* < 0.01, *** *p* < 0.001.

We further investigated the effect of HSF2BP on the proliferation of LUAD cells. The protein expression of HSF2BP was significantly upregulated in NCI-H209 and A549 cells. Thus, the NCI-H209 and A549 cells were infected with sh-HSF2BP. The HSF2BP knockdown efficiency in NCI-H209 and A549 cells after lentivirus infection was confirmed by western blot analysis (Figure 3a). Then, CCK-8 assays were performed to confirm whether the downregulation of HSF2BP affects the proliferation ability of LUAD cells. As shown in Figure 3b, the HSF2BP accelerated the growth of LUAD cells. In addition, the HSF2BP knockdown drastically reduced the colony-forming capacity of NCI-H209 and A549 cells in a colony formation assay. These results demonstrated that HSF2BP promoted the proliferation of LUAD cells (Figure 3c).

### 3.3. HSF2BP promoting the migration of LUAD cells

The effect of HSF2BP on the migration of LUAD cells was investigated using a wound-healing experiment. Results showed that the wound healed faster in A549/shCtrl and NCI-H209/shCtrl cells than in HSF2BP knockdown cell lines (Figure 3d). To further confirm the effects of HSF2BP on LUAD migration, a transwell assay was performed. As shown in Figure 3e, the HSF2BP knockdown cells led to a reduced number of migrated cells. These results indicate that HSF2BP serves as an oncogene of LUAD.

### 3.4. HSF2BP involvement in the regulation of the MAPK signaling pathway

**Figure 4.**
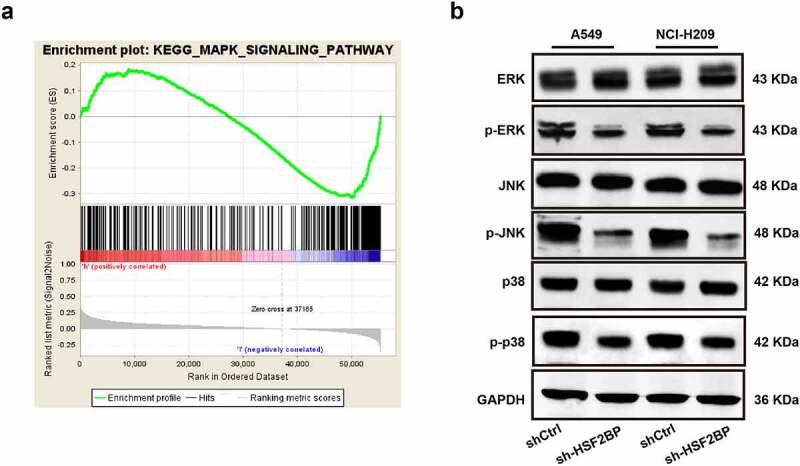
HSF2BP is involved in the regulation of MAPK signaling pathway. (a) GSEA analysis of HSF2BP. (b) The expression level of p-ERK, total ERK, p-JNK, total JNK, p-p38 and total p38 in LUAD cells by western blot.

To further explore the potential mechanism of HSF2BP in regulating the progression of LUAD, a GSEA was conducted based on TCGA datasets. Results showed that HSF2BP was enriched in the MAPK signaling pathway (Figure 4a). Hence, to assess the total protein level and phosphorylation levels of ERK, NK, and p38, a western blot analysis was conducted. The blots revealed that the expression of ERK, JNK, and p38 phosphorylation decreased in the HSF2BP knockdown group, while the total ERK, JNK, and p38 levels were not different (Figure 4b). These results indicated that HSF2BP can promote the progression of LUAD by regulating the MAPK signaling pathway.

### 3.5. HSF2BP promoting LUAD cells tumorigenesis in vivo

**Figure 5.**
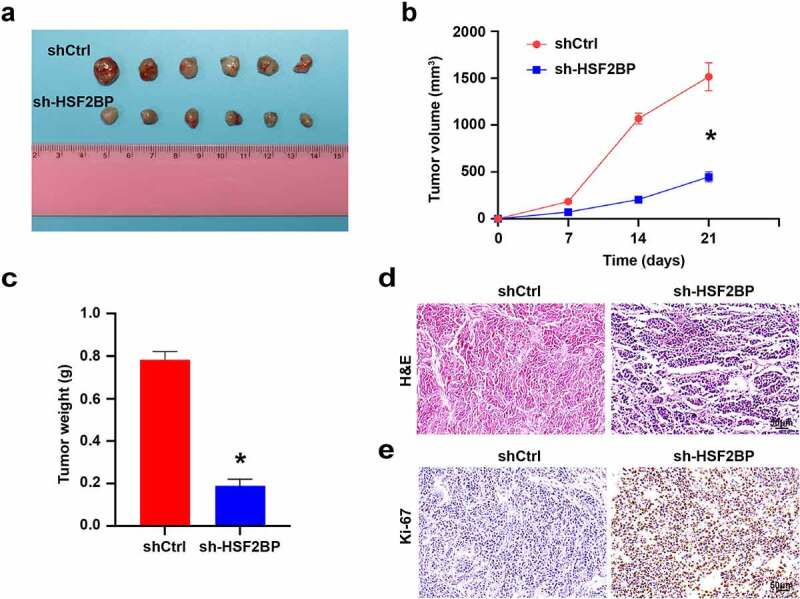
HSF2BP promotes the tumor growth *in vivo*. (a) The tumor formed by shCtrl and sh-HSF2BP cells. (b) Tumor volume. (c) Tumor weight. (d) HE staining. (e) Ki67 expression detected by immunohistochemistry. **Note**: * *p* < 0.001.

To investigate how HSF2BP affects the progression of LUAD *in vivo*, we carried out the xenograft models by A549. HSF2BP knockdown inhibited the tumor formation, tumor growth rate and tumor volume (Figure 5a-c). In addition, HE staining and IHC were used to detect the expression of Ki67 in xenografts tumors. HE staining confirmed the presence of tumor (Figure 5d). Compared with shCtrl group, Ki67 expression was down-regulated in the sh-HSF2BP group (Figure 5e), which indicated that HSF2BP knockdown inhibited the tumor growth *in vivo*.

## 4. Discussion

This study identified HSF2BP as a target gene involved in the LUAD cell proliferation and migration. The evidence indicated that HSF2BP was overexpressed in LUAD cell lines and tissues compared with that in the adjacent tissues. Furthermore, HSF2BP could act as an independent prognostic biomarker for LUAD. Mechanistically, GSEA revealed that HSF2BP might be associated with the activation of the MAPK signaling pathway. We speculated that HSF2BP may reinforce the proliferation and migration of cells, which is due to the activation of the MAPK pathway.

Previous studies have shown that HSF2BP, which regulates meiosis, is associated with genomic instability. HSF2BP missense mutations may be a potential molecular mechanism of human ovarian dysfunction [16]. In addition, HSF2BP interferes with DNA damage repair by binding to BRCA2 [14]. Genomic instability is one of the hallmarks of cancer [17]. Therefore, HSF2BP is expected to be a promising target for cancer treatment. To date, the expression and biological function of HSF2BP in tumor tissues have not been specifically studied. Here, we studied the expression of HSF2BP in patients with LUAD based on the TCGA database and determined that it is overexpressed in LUAD compared with that in normal tissues, which is consistent with the qRT-PCR results. The high expression of HSF2BP is positively correlated with tumor stage, indicating that HSF2BP has an important effect on the progression of LUAD. The Kaplan-Meier analysis and Cox regression analysis showed that the upregulation of HSF2BP was associated with poor prognosis, indicating that HSF2BP might be a prognostic predictor. In vitro functional experiments confirmed that HSF2BP enhanced the proliferation and migration of LUAD cells, which has not been previously reported, suggesting that HSF2BP plays a role in the development of LUAD. We further validated that HSF2BP promoted the proliferation of LUAD cells *in vivo*.

The MAPK pathway is hyperactivated in human cancers; ERK, JNK, and p38, known as elements of the MAPK pathway, are considered as oncogenes that promote cell proliferation, differentiation, angiogenesis, and migration [18,19]. Patients with LUAD showed an abnormal activation of the MAPK pathway and had more frequent KRAS mutations [20,21]. As a component of the MAPK pathway, KRAS mutations are associated with a poor prognosis of LUAD. In recent years, inhibitors targeting the MAPK pathway have been frequently used in patients with LUAD, which improved the survival rates of patients [22]. Targeting the MAPK pathway could improve the efficacy of immunotherapy in LUAD by inducing the expression of PD-L1 [23]. To explore the molecular mechanism by which HSF2BP exerts its effects on LUAD, GSEA was performed based on the data obtained from the TCGA database. The GSEA showed that the MAPK pathway was positively correlated with HSF2BP expression. Furthermore, the HSF2BP knockdown decreased the phosphorylation of ERK, JNK, and p38, which was verified by western blot analysis. Therefore, we hypothesized that HSF2BP could promote LUAD cell proliferation and migration via the MAPK signaling pathway. However, considering the effects of HSF2BP on meiosis and DNA damage repair, the HSF2BP might also regulate the progression of LUAD. However, further study is required to determine the specific regulatory mechanisms.

## 5. Conclusion

Above all, HSF2BP is overexpressed in LUAD tissues and positively associated with lower overall survival in LUAD patients. HSF2BP enhances the malignant biological behavior of LUAD, which may be attributed to the activation of MAPK downstream elements. Thus, HSF2BP is expected to be a promising target for the treatment of human LUAD.

## Data Availability

All data are available from the corresponding author on reasonable request.

## Highlights

- Lung adenocarcinoma (LUAD) is the most frequently diagnosed histological subtype.
- HSF2BP is involved in the LUAD cell proliferation and migration.
- HSF2BP is a promising target for the treatment of human LUAD.
